# Transoral Robotic Surgery for Head and Neck Cancer: Advances and Residual Knowledge Gaps

**DOI:** 10.3390/jcm12062303

**Published:** 2023-03-16

**Authors:** Mariam H. Mella, Emilien Chabrillac, Agnès Dupret-Bories, Mathilde Mirallie, Sébastien Vergez

**Affiliations:** 1 Department of Otolaryngology, Head and Neck Surgery, Toulouse University Hospital—Larrey Hospital, 24 Chemin de Pouvourville, CEDEX 9, 31059 Toulouse, France; 2 Department of Surgery, University Cancer Institute of Toulouse—Oncopole, 31100 Toulouse, France

**Keywords:** transoral robotic surgery, TORS, head and neck cancer, squamous cell carcinoma, minimally invasive surgery

## Abstract

Minimally invasive surgery is a growing field in surgical oncology. After acquiring its first Food and Drug Administration approval in 2009 for T1–T2 malignancies of the oral cavity, oropharynx, and larynx, transoral robotic surgery (TORS) has gained popularity thanks to its wristed instruments and magnified three-dimensional view, enhancing surgical comfort in remote-access areas. Its indications are expanding in the treatment of head and neck cancer, i.e., resection of tumors of the larynx, hypopharynx, or parapharyngeal space. However, this expansion must remain cautious and based on high-level evidence, in order to guarantee safety and oncological outcomes which are comparable to conventional approaches. This narrative review assesses the current role of TORS in head and neck cancer from an evidence-based perspective, and then identifies what knowledge gaps remain to be addressed.

## 1. Introduction

Head and neck cancer (HNC) incidence has increased in the past decades, ranking eighth among the most common cancers worldwide in 2020 with approximately 900,000 cases yearly [1]. The main risk factors for head and neck cancer include smoking, alcohol abuse, and human papillomavirus (HPV). HPV has become a prominent risk factor, being responsible for 12–63% of oropharyngeal cancers in the past two decades globally [2,3].

Treatment modality is determined by tumor location, stage, and patient comorbidities. Advances in the fields of surgery, chemothepy, and radiotherapy (RT) have a dual goal of ensuring the best oncological outcomes while preserving quality of life. Despite their adequate oncological outcomes, traditional therapeutic options for HNC place a burden on functional results. Thus, it is by improving the postoperative course and functional outcomes that minimally invasive surgery has gained recognition in the field of HNC [4]. 

Robotic surgery using the Da Vinci^®^ surgical system gained popularity in the early 2000s in the fields of urology and gynecology thanks to its magnified three-dimensional vision, absence of surgeon tremor, and higher precision. This technology offers better surgical comfort in remote-access areas due to the presence of wristed instruments with seven degrees of freedom [4]. In 2007, the concept of transoral robotic surgery (TORS) was introduced by Weinstein et al. [5]. Their study on radical tonsillectomy inspired further research in the field and, finally, in 2009, TORS received Food and Drug Administration (FDA) approval for resections of T1–T2 malignancies of the oral cavity, pharynx, and larynx, as well as benign lesions [5,6,7,8]. Currently, with further technological advances, as well as the need for minimally invasive surgery and increased surgical experience, TORS indications have continued to expand and now include resection of tumors of the larynx, hypopharynx, parapharyngeal space, and skull base [9], as well as neck dissection and thyroidectomy, which fall outside the scope of this TORS review [10,11,12]. 

The aim of this narrative review is to assess the current role of TORS in HNC from an evidence-based perspective, and to identify what knowledge gaps remain to be addressed.

## 2. Preoperative Considerations and Eligibility Criteria

Patient selection is driven by various criteria that determine whether a patient is eligible for TORS procedure or has to pursue conventional therapy. This set of criteria can be divided into three main parts: medical history, imaging/tumor extension, and surgical exposure.

### 2.1. Medical History

Like for any type of surgery, medical comorbidities need to be assessed in order to predict possible complications and correct reversible states prior to surgery. Congestive heart failure, pulmonary disease, connective tissue diseases, morbid obesity, poorly controlled diabetes, immunosuppression, and malnutrition are considered as relative contraindications for surgery [4,13]. In addition to determining the patient’s operability, these comorbidities also weigh on the decision to perform a prophylactic tracheostomy [14,15].

### 2.2. Imaging/Tumor Extension

Each anatomical subsite has specific features which make it eligible for TORS or not, due to tumor extension and/or relation to anatomical structures. An optimal pretherapeutic workup with computed tomography (CT) and/or magnetic resonance imaging (MRI) is paramount. While a CT scan can be sufficient for laryngeal/hypopharyngeal cancer staging, MRI is essential for the staging of tumors arising or extending above the hyoid bone [16]. Tumor extension must allow a surgical excision with safety margins. As per a recent meta-analysis, involved margins are found in 8.1% of TORS oropharyngectomies, with a negative impact on local control [17]. The ideal margin cutoff to regard as a clear resection margin has not been well defined to date [17]. A recently published multicentric cohort studied the effect of margin dimensions on local control in TORS; it showed that, for an adequate local control, 1.1 mm margins of healthy tissue around the tumor are sufficient [18].

Tonsillar cancer: The absolute contraindications for tonsillar cancer resection include internal carotid artery encasement, involvement of the mandibular periosteum, prevertebral fascia/musculature, or masticator space musculature. Relative contraindications also exist, in which case feasibility to carry out surgery also depends on surgical experience with the robotic system. These include involvement of the pre-styloid parapharyngeal space, hard palate, and extension into the nasopharynx. A medialized/retropharyngeal internal carotid artery is an anatomical variation that can preclude one from performing TORS safely and achieving sufficient margins [19].

Base-of-tongue cancer: Relative contraindications for excision include invasion of the hyoglossus muscle, which would place the hypoglossal nerve at risk during resection. In this case, an open approach would provide better functional outcomes with lower risk of hypoglossal nerve injury. Another significant, yet not absolute, contraindication is extensive invasion of the genioglossus muscle due to its risk of lingual artery injury laterally during surgery. Tumor proximity with both lingual arteries during surgery places the tongue at risk of devascularization [20]. Of note, landmarks have been described for the identification of the lingual artery and hypoglossus nerve during transoral surgery [21,22]. Furthermore, infiltration of the pre-epiglottic fat and extension close to the hyoid bone could pose a challenge to achieving negative margins; therefore, these are relative contraindications for TORS [20].

Laryngeal cancer: Tumors resectable with TORS should not invade the thyroid or cricoid cartilages, pre-epiglottic space, paraglottic space, posterior commissure, base-of-tongue musculature, or more than 20 mm of base-of-tongue mucosa [4,20].

Hypopharyngeal cancer: There are limited studies of TORS use within this anatomical subsite; however, eligible patients would be those with superficial tumors that respect the apex and lateral wall of the pyriform sinus (due to the underlying thyroid cartilage) and that have neither bony nor cartilaginous involvement [20,23].

### 2.3. Surgical Exposure

Independent of the disease extension, an inadequate exposure is a contraindication for TORS. As for transoral laser microsurgery, TORS demands a specific evaluation of the surgical site exposure. This evaluation is frequently performed during a panendoscopy, where the robotic mouth gag should be tried by a surgeon experienced in TORS. It includes six “Ms” for TORS: microstomia, micrognathia, mandibulo-maxillary abnormalities, macroglossia, restricted cervical mobility, and mouth opening. Any of these factors could limit surgical exposure, rendering TORS surgery unfeasible. Another “M” factor that could also affect exposure is morbid obesity; however, it is not an absolute contraindication [24].

Morphometric measurements have been proven to significantly predict exposure quality, e.g., mandibular body height, hyoid–mental distance, and neck circumference [25]. A large prospective study showed that independent risk factors for difficult oropharyngeal exposure were modified Mallampati class ≥III, small inter-incisor gap, and large neck circumference, which led the authors to propose a score specific to oropharyngeal exposure [26]. Similarly, imaging-based measurements can predict access to the base-of-tongue tumors. Three factors proved to be statistically significant i.e., distance from posterior pharyngeal wall to hyoid (≤30 mm), angle between the epiglottis and vertical plane of the larynx (≥130°), and distance from the posterior pharyngeal wall to the soft palate (≤8.1 mm) [27].

### 2.4. Surgeon Expertise

Surgeon expertise is another factor influencing surgical exposure and surgical outcomes in TORS. The introduction of TORS into daily practice requires a certain level of training and expertise that is usually measured by operative time and margin status. Without sufficient evidence, a consensus between the main academic institutions including the American Head and Neck Society (AHNS) Education Committee, American Academy of Otolaryngology—Head and Neck (AAO-HNS) Surgery Robotic Task Force, AAO-HNS Sleep Disorders Committee, and the ECOG 3311 trial proposed a minimum of 20 cases to attain TORS accreditation [28]. Some authors retrospectively studied the learning curves of three surgeons exclusively performing TORS for oropharyngeal cancer over 6 years, suggesting that most surgeons have inflection points between 20 and 30 cases [29].

## 3. TORS Applications in HNC

Despite its acknowledgement worldwide, TORS use in HNC is not recommended globally. Developing countries (e.g., Brazil and African or Asian countries) battle with the cost and expertise required for the use of TORS, whereas the British National Health Service (NHS) argues that there is insufficient evidence to make this treatment routinely available [30]. Likewise, in Germany, there are no data supporting an official implementation of TORS, although it is used by 21.4% of university medical centers, arguing that transoral approaches can be carried out with the help of a laser. Reasons explaining the low adoption rate of TORS in Germany (0.8%) are its cost, the lack of cost-covering reimbursement, and insufficient cooperation with other disciplines and hospital administration [31]. Indeed, the cost-effectiveness of TORS remains unknown in many indications, and, while it is under scrutiny in the United States, it has hardly been studied in Europe [32]. The Nordic countries (Denmark, Finland, Iceland, Norway, and Sweden) suffer from a scattered TORS availability in the region. Specifically, 10/21 HNC centers perform TORS; however, each center operates on fewer than 25 cases annually, and 9/10 centers report a high complication rate [33]. On the other hand, a late study showed that, between 2010 and 2016, the use of TORS for oropharyngeal cancer treatment in the United States nearly doubled [34]. Following a similar trend, head and neck surgeons in Australia and New Zealand have a TORS adoption rate of 43.6% [35]. The main indications for TORS in all these countries are lateral oropharyngectomy, base-of-tongue mucosectomy, and surgery for obstructive sleep apnea.

### 3.1. Oropharyngeal Cancers

Oropharyngeal squamous cell carcinomas (SCCs) amenable to excision by TORS are mainly T1 and T2 tumors. Selected T3 (size ≈ 4–5 cm) and T4a (limited invasion or the stylo-glossus muscle) can also be treated with TORS; however, the rate of positive margins significantly increases with T-status, i.e., 13%, 17.1%, 28.2%, and 45.9% for T1, T2, T3, and T4a tumors, respectively [36,37].

Although the historical standard treatment of early-stage oropharyngeal SCC was RT, retrospective studies have validated the use of TORS in a bid to de-escalate treatment, especially for HPV-positive SCC [38]. The arguments in favor of upfront surgery are its ability to provide accurate tumor staging, and the possibility to spare adjuvant radiotherapy in selected cases (pN0–pN1 with clear margins). In practice, many patients treated with TORS + neck dissection require postoperative RT, and indications for single modality treatment with surgery are scarce [39]. Lastly, TORS is cost-effective for early-stage oropharyngeal cancer. It demonstrated cost savings of 1366 USD and an increase of 0.25 quality-adjusted life years per case in comparison to (C)RT [40].

A multicentric cohort by de Almeida et al., the largest so far, studied 410 patients with head and neck SCC treated with TORS with or without postoperative RT or chemoradiotherapy (CRT). The vast majority had oropharyngeal SCC (88.8%) and early-stage disease (83.5%). The 3 year overall survival (OS) and disease-specific survival (DSS) were 87.1% and 94.5%, respectively, comparable to the standard of care [38]. A systematic review of 772 patients with early oropharyngeal SCC corroborated these data and demonstrated that the 2 year survival ranged between 82% and 94% [41].

Studies comparing TORS outcomes in patients with oropharyngeal p16-positive and p16-negative SCCs have demonstrated a relatively poor prognostic significance of HPV status in terms of survival [38,42]. In their study of 57 patients with HPV-negative oropharyngeal SCCs, Dabas et al. observed a locoregional control, DFS, and OS of 95.8%, 89.6%, and 93.8% after a mean follow-up of 29 months [24]. On the other hand, in a cohort of 48 HPV-positive oropharyngeal SCCs treated with TORS, the 5 year locoregional control, DSS, and OS were 98%, 100%, and 95% (Table 1) [2].

The results of the phase II clinical trial ORATOR were published recently, in which patients with early-stage OPSCC were randomized into two arms, i.e., TORS and neck dissection ± postoperative (C)RT versus definitive (C)RT. Outcomes of swallowing-related quality of life were better in the definitive (C)RT arm after 1 year, although the difference was not clinically meaningful and decreased over time (Table 2). Of note, trismus was more common in the TORS arm (26% vs. 3%), and dry mouth scores were higher in the RT arm. Lastly, pain and dental-related issues were more common in the TORS arm [43]. The conclusion of this phase II study was that TORS + neck dissection and definitive (C)RT have comparable toxicity profiles, and that patients with early-stage oropharyngeal SCC should be informed of both treatment options. The main limitation of this study is that, among 34 patients in the TORS + neck dissection arm, 71% had postoperative (C)RT, notably because of their advanced nodal stage (53% of cN2 patients). Therefore, the question of whether RT is more toxic than TORS alone in that indication remains unanswered. 

Upfront TORS is also an option for selected stage III/IV oropharyngeal SCC to intensify therapy, especially in HPV-negative oropharyngeal SCC [44]. When followed by (C)RT, TORS has proved to improve survival outcomes [45]. This is in keeping with observations outside the context of TORS, where upfront surgery followed by adjuvant (C)RT results in better survival than definitive (C)RT [46,47]. In a recent retrospective study of 136 patients with HPV-positive SCCs, Zebolsky et al. showed that upfront TORS + neck dissection was an appropriate first line treatment for cN0–cN2a cases without clinical signs of ENE, in order to reduce postoperative RT dose/extent and/or avoid adjuvant chemotherapy. Pathologic ENE was present in 35.6% of cN2b patients, with a threefold higher likelihood compared to cN1–cN2a patients [45,48].

### 3.2. Carcinomas of Unknown Primary

After a negative workup and negative palatine tonsillectomy, performing a base-of-tongue mucosectomy (lingual tonsillectomy) with either TORS or transoral laser microsurgery could detect the primary tumor in about half of cases [6,49,50,51,52]. However, the recommendations of the main academic societies differ significantly and evolve rapidly as knowledge in the field progresses. Given the consequences on treatment initiation delay, the morbidity of this procedure, and the variable availability of TORS, the American Society of Clinical Oncology (ASCO) guidelines state that indication for base-of-tongue mucosectomy and its laterality remain at the discretion of the surgeon [53]. The National Comprehensive Cancer Network (NCCN) guidelines support base-of-tongue mucosectomy after a negative palatine tonsillectomy. Of note, they advise against bilateral palatine and lingual tonsillectomy due to the risk of circumferential oropharyngeal stenosis [54]. On the contrary, the AHNS advocates for simultaneous bilateral palatine tonsillectomy with ipsilateral base-of-tongue mucosectomy, due to allegedly high rates of finding a primary in the contralateral palatine tonsil (15–25%) and in the ipsilateral lingual tonsil (6%) [52]. However, a recent systematic review challenged this as it highlighted a lower rate of contralateral tonsillar primary, i.e., 10% and 1% of bilateral and contralateral tonsillar primary, respectively [55]. These numbers likely vary as the prevalence of HPV-associated SCC varies between countries. This variation may, therefore, justify the global difference of practices and recommendations regarding palatine and/or lingual tonsillectomy.

With respect to the HPV status, the likelihood of finding the primary tumor in a base-of-tongue mucosectomy in HPV-negative carcinomas of unknown primary seems much lower than in their HPV-positive counterparts (13% versus approximately 50%), and most studies to date included mainly HPV-positive patients [6,56,57]. Therefore, in HPV-negative carcinomas of occult primary, the risk/benefit balance of base-of-tongue mucosectomy seems currently unfavorable.

### 3.3. Laryngeal Cancers

Most indications for TORS excision of laryngeal SCC are early-stage (T1–T2 N0–N1) supraglottic SCCs. However, TORS also allows performing cordectomies for early glottic cancers, as well as total laryngectomies for more advanced tumors. Similarly to transoral laser microsurgery, the rationale for using TORS in early laryngeal tumors is to carry out a single modality treatment and to avoid the morbidity of external approaches. Indeed, TORS can provide favorable functional outcomes thanks to the preservation of key structures for swallowing, e.g., infrahyoid muscles, pharyngeal constrictor muscles, hyoid bone, thyroid cartilage, and superior laryngeal nerve. This results in fewer prophylactic tracheostomies, a faster recovery time and return to oral feeding, lower risk of aspiration pneumonia, and a shorter hospital stay [58].

Supraglottic laryngectomy: After the first use of TORS for supra-glottic laryngectomy in a canine model in 2005, this technique has spread widely and has now become a routine therapeutic option for supraglottic cancer [4]. However, supraglottic cancers are rarely diagnosed at an early stage, and surgical exposure is the main limiting factor for their excision by TORS. A recent systematic review by Lechien et al. studied the outcomes of TORS supraglottic laryngectomy in 422 patients. The majority of cases were cT2 (48.6%), followed by cT1 (35.8%) and cT3 (5.1%). Tumors involved the epiglottis, aryepiglottic fold, or false vocal cords in 55.4%, 31.2%, and 5.1% of cases, respectively [59]. Three studies reported conversion to open surgery despite adequate preoperative evaluation of the exposure: 2.1% for Dabas et al., 4.4% for Lallemant et al., and 20% for Ansarin et al. [60,61,62].

Oncological outcomes of TORS supraglottic laryngectomies appear satisfactory, although no randomized controlled study has compared it to open partial laryngectomies or (C)RT to date. All comparisons are retrospective and suffer from a selection bias. In Lechiens’s systematic review, the rate of positive margins was 5.4% and ranged from 0% to 40% [62]. In all series from this review, the 2 year local and regional control rates exceeded 94.3% and 87.5% respectively. The 2 year OS ranged between 66.7% and 88.0%, with a distant metastasis rate of about 9%. In the largest series published to date, OS and disease-free survival (DFS) were 86.9% and 95.1% at 2 years, and 78.7% and 94.3% at 5 years, respectively (Table 1). Of note, half of them required adjuvant radiotherapy [63]. 

Postoperative outcomes of TORS supraglottic laryngectomies are favorable. Contrarily to open partial laryngectomy, the rate of tracheotomy is low in TORS supraglottic laryngectomy. In a French series of 84 patients, 24% of patients had a tracheostomy, for a median period of 8 days. Only one patient could not be decannulated during follow-up [64]. Prophylactic tracheostomies were performed to improve the surgical exposure or to secure the airway in case of postoperative edema or bleeding, which peak at 48 h post TORS [65].

With respect to functional outcomes, oral diet was resumed on day 37 by Weinstein et al., on day 8 by Park et al. [66], and on day 1 by Ozer [59]. Razafindranaly et al. used a nasogastric tube in 76% of patients, for a median period of 8 days. Almost 10% of patients required a definitive gastrostomy. Few studies have analyzed speech outcomes after TORS. In their prospective study of 16 patients who underwent TORS supraglottic laryngectomy, Park et al. reported a postoperative voice handicap index (VHI) at 15 months below 20 in 62.5% of patients, and between 20 and 40 in 31.3% of cases [66]. Similarly, a retrospective study indicated that normal speech and mild and moderate dysphonia were present in three, two, and four patients out of nine, respectively [67].

Total laryngectomy: Future developments of TORS include total laryngectomy. Its theoretical advantages are the preservation of pre-laryngeal soft tissue and muscle, which could decrease the risk of fistula and avoid the need for flap coverage. Its main drawbacks are the difficult exposure and the extended operative time, estimated around 300 min in the largest series published [68]. Its indications are restricted to situations where a neck dissection is not indicated, i.e., selected cases of salvage laryngectomy for small endolaryngeal tumors, a nonfunctional larynx, or histologies with limited potential for lymphatic spread, e.g., chondrosarcoma and adenoid cystic carcinoma [4]. For all these reasons, only five case series of TORS total laryngectomy have been published until today [68,69,70,71,72].

The latest and largest case series included 10 patients. Excision margins were negative in all cases. Two fistulas (20%) and one minor postoperative hemorrhage occurred. Time to oral feeding resumption ranged from 6 to 24 days [68]. The small size of published cohorts prevents drawing a clear comparison with the standard of care.

### 3.4. Hypopharyngeal Cancers

Few studies in the literature have reported outcomes of TORS hypopharyngectomy; however, these are promising [73]. The largest study published to date is that of Mazerolle et al., in which 57 patients underwent TORS for T1–T2 pyriform sinus SCC. After 2 years of follow-up, the OS and DFS rates were 84% and 74%, respectively. After 4 years, they were 66% and 50% [23]. Another team reported favorable outcomes of TORS hypopharyngectomy ± adjuvant treatment in 22 patients. Patients started oral feeding on average 7 days postoperatively and were discharged after a median period of 13 days. The 5 year DSS, DFS, and OS were 91.7%, 57.1%, and 53.7%, respectively [74]. TORS with simultaneous neck dissection was performed in 38 patients, in a study by Park et al., where the DFS at 5 years was 100% for early-stage disease and 68.6% for late-stage disease (Table 1) [75].

Therefore, TORS shares the survival benefits of surgery over concomitant (C)RT, but with better functional outcomes than open surgery [73]. In the absence of a consensus regarding the treatment of hypopharyngeal SCC, TORS is a viable single modality therapeutic option for selected T1–T2 N0–N1 hypopharyngeal tumors. However, such indications are rare, and difficulties of exposure are frequent; therefore, this approach requires a high experience in TORS. Future developments of flexible single-port robotic systems may extend indications of TORS hypopharyngectomy [76].

**Table 1:** Oncologic outcomes of transoral robotic surgery.

	N (Patients)	Follow-Up	DSS	DFS	OS	Loco-Regional Control
Oropharyngeal SCC						
De Almeida (2015) [38]	410	2–3 years	94.5–92.5%	-	91–87.1%	91.8–88.8%
Dabas * (2017) [45]	57	29 months	-	89.6%	93.8%	95.8%
Nichols ** (2021) [39]	48	2.5 years	100%	-	95%	98%
Supraglottic SCC						
Lechien (2020) *** [60]	422	5 years	-	94.3%	78.7–80.2%	87.7–89.2%
Doazan (2018) [64]	122	42.8 months	-	94.3%	78.7%	90.2%
Hypopharyngeal SCC						
Mazerolle (2018) [23]	57	4 years	-	50%	66%	-
Park (2017) [77]	38	5 years	Stage I/II: 100%	Stage I/II: 100%	-	-
			Stage III/IV: 74%	Stage III/IV: 68.6%		
Hassid (2020) [76]	22	5 years	-	57.10%	53.10%	-

* HPV-negative; ** HPV-Positive; *** Systematic review. DSS: disease specific survival; DFS: disease free survival; OS: overall survival; SCC: squamous cell carcinoma.

**Table 2:** ORATOR clinical trial: Long term swallowing outcomes.

	MDADI at 1 Year	MDADI at 2 Years	MDADI at 3 Years
Radiotherapy	86.9 ± 11.4	86 ± 13.5	88.9 ± 11.3
TORS + ND	80.1 ± 13	84.8 ± 12.5	83.3 ± 13.9
*p*-value	0.049	0.74	0.12

MDADI: MD Anderson Dysphagia Inventory.

### 3.5. Retropharyngeal Neck Dissection

Retropharyngeal lymph nodes are a common metastatic site for some head and neck cancers, and their involvement often leads to the disease being considered unresectable [77]. However, TORS has been used to treat retropharyngeal node metastasis instead of RT or as an adjunct, in order to reduce RT toxicity and to provide a reliable staging that could guide treatment [10,78]. It is especially useful in cancers for which the standard treatment is surgery, e.g., retropharyngeal metastasis of papillary thyroid carcinoma [4,10,78,79]. While the literature about TORS for retropharyngeal node dissection is too scarce to define its potential role and indications, it is clear that TORS can provide adequate access to the retropharyngeal space [79]. The ideal indication may be an isolated retropharyngeal node <3 cm in size [4].

### 3.6. Parapharyngeal Space Surgery

The parapharyngeal space is a deep space of the face with a complex anatomy. It contains major vasculature which poses a surgical risk. According to tumor characteristics and local extension, the approach can be transoral and/or transparotid–transcervical [80]. TORS can be used for selected well-defined tumors of the parapharyngeal space with oropharyngeal bulge. Surgical exposure depends on mouth opening and tumor extension. Contraindications for TORS are medialization of the internal carotid artery by the mass effect, tumor extension past the stylomandibular ligament, or location less than 1 cm from the skull base. 

A significant drawback of TORS is the absence of haptic feedback, which makes it difficult to feel tumor extensions. This is why surgeons often resort to finger palpation in the midst of a TORS. Major vessel injury during a transoral procedure is likely to result in a conversion into open surgery for ligation. Other complications include dehiscence of the pharyngeal incision, nerve injury, or rupture of the tumor [80,81,82]. Of note, capsular breach of salivary gland tumors, possibly due to lack of palpation, has been reported to be higher via TORS than via open approaches [83].

## 4. Postoperative Course and Complications

In general, TORS complication rates are reportedly lower than those of open approaches. However, they can be life-threatening. Indeed, post-TORS fatality has been reported between 0.3% and 1.1% [84]. 

The most dreaded complication is postoperative hemorrhage, ranging between 1.5% and 18.5% [85]. Minor hemorrhages are more common than major ones, and they are often managed conservatively. In only 2.9% of TORS cases, an invasive technique is needed to control the bleeding i.e., emergent embolization, tracheostomy, or transcervical arterial ligation [86,87]. Thanks to several meta-analyses, prophylactic ligation of external carotid artery branches during concomitant neck dissection has become the standard of care for TORS oropharyngectomy. It decreases the risk of major and severe hemorrhage, defined as bleeding that requires ligation/embolization and life-threatening bleeding, respectively. However, it does not seem to prevent minor hemorrhage [85,87]. Conversely, arterial ligation does not seem to provide the same benefit for TORS supraglottic laryngectomy. Explanations are that the associated overall risk of hemorrhage is lower (3.7%) since the laryngeal vascularization is less abundant, with fewer anatomical variations, and is readily amenable to transoral ligation or coagulation [86].

Airway-related complications remain understudied. However, according to a meta-analysis, their incidence in TORS supraglottic laryngectomy is 4.92%, with 3.38% of emergent tracheostomy for airway compromise [86]. Indeed, TORS for HNC does not systematically require a tracheostomy. According to a French expert survey, high-risk situations such as previous radiation therapy with residual edema or anticoagulation therapy with large resections might be indications for preventive tracheostomy [14,15].

Additional complications, yet less frequent, include neck hematoma in 0.4% and pharyngo-cutaneous fistula in 2.5% of cases [38,87,88]. Spondylodiscitis is a severe complication to keep in mind after TORS for SCCs of the posterior pharyngeal wall, occurring after a mean interval of over 2 months postoperatively [89]. Complications related to robot docking and instrument collision include lip, dental, and corneal injury. Lingual paresthesia due to lingual nerve injury or tongue compression by the mouth gag, as well as hypoglossal nerve injury, has also been reported [84]. Patients older than 65 years notably develop more complications as opposed to the younger population, making this a relevant risk factor to be assessed when choosing candidates for TORS [13].

As experience with TORS increases, reductions in operative time, length of intubation, hospital stay, and positive margin rate have been highlighted [29,90]. These could be explained by a faster docking, better patient selection, and better perioperative management (e.g., prophylactic tracheostomy) [58]. Experienced surgeons may also compensate for the lack of haptic feedback with the interpretation of visual cues such as color change when pressing.

## 5. Technological Advances in TORS

The development of the Da Vinci^®^ single-port (SP) robot, designed initially for urology, has proved advantageous for TORS. Not only can three robotic arms be inserted through the 2.5 cm single port, but the endoscope arm itself can be turned into a “cobra head” to obtain a better exposure. This provides optimal visualization for the hypopharynx and larynx [76]. The docking time is shorter, conflicts of instruments are limited. and maneuverability is improved [91]. Consequently, the SP system seems to have a faster learning curve and shorter operative times while maintaining the functional and oncological outcomes associated with Si and Xi systems [76,92,93].

Another lead to improve surgical comfort and efficiency is the development of improved mouth gags and fine micro-instruments designed to meet the needs of the HNC surgeon. Indeed, no instruments have yet been developed specifically for TORS; thus, instruments tend to be oversized. Furthermore, with the introduction of the SP system, Intuitive Surgical has stopped the manufacturing of the 5 mm micro-instruments for the Da Vinci^®^ X and Xi systems that were initially used in TORS with the Si system. 

## 6. Conclusions

TORS has provided a breakthrough in the field of minimally invasive treatment of HNC. However, because robotic surgery was initially developed for urological and gynecological procedures, it lacks some adequate functionalities that would allow its wider use in the field of HNC. The newer SP model has already begun to provide technical advantages in remote areas such as the hypopharynx and larynx. If technical advances continue to tailor the DaVinci^®^ robotic system to suit the complex anatomy of the head and neck, indications and global acceptance will continue to expand, and the surgeon’s learning curve will shorten. It is obvious that, with the rapidly advancing technology, this goal is not far away. For now, however, it is with further experience that we will achieve better surgical outcomes.

## Data Availability

Data sharing not applicable. No new data were created or analyzed in this study. Data sharing is not applicable to this article.
